# Recommendations for the Diagnosis, Prevention, and Control of Coronavirus Disease-19 in Children—The Chinese Perspectives

**DOI:** 10.3389/fped.2020.553394

**Published:** 2020-11-05

**Authors:** Feng Fang, Yu Chen, Dongchi Zhao, Tonglin Liu, Yongjian Huang, Liru Qiu, Yan Hao, Xiaolin Hu, Wei Yin, Zhisheng Liu, Runming Jin, Qin Ning, Pik-to Cheung, Chunfeng Liu, Sainan Shu, Tianyou Wang, Xiaoping Luo, The Chinese Pediatric Society and the Editorial Committee of the Chinese Journal of Pediatrics

**Affiliations:** ^1^Department of Pediatrics, Tongji Medical College, Tongji Hospital, Huazhong University of Science and Technology, Wuhan, China; ^2^Department of Pediatrics, Zhongnan Hospital of Wuhan University, Wuhan, China; ^3^Tongji Medical College, Wuhan Children's Hospital, Huazhong University of Science, Wuhan, China; ^4^Department of Pediatrics, Tongji Medical College, Union Hospital, Huazhong University of Science and Technology, Wuhan, China; ^5^Department of Infectious Disease, Tongji Hospital of Tongji Medical College, Huazhong University of Science and Technology, Wuhan, China; ^6^Virtus Medical Group, Pediatric Endocrinology, Genetics, and Metabolism, Hong Kong, China; ^7^Department of Pediatrics, Shengjing Hospital, China Medical University, Shenyang, China; ^8^Beijing Children's Hospital, Capital Medical University, Beijing, China

**Keywords:** COVID-19, SARS-CoV-2, pandemic (COVID-19), children, consensus recommendations, guidelines

## Abstract

Ever since SARS-CoV-2 began infecting people by the end of 2019, of whom some developed severe pneumonia (about 5%), which could be fatal (case fatality ~3.5%), the extent and speed of the COVID-19 outbreak has been phenomenal. Within 2.5 months (by March 18, 2020) over 191,127 COVID-19 patients have been identified in 161 countries. By then, over 700 pediatric patients were confirmed to have COVID-19 in China, with only about 58 diagnosed elsewhere. By now, there are thousands of children and adolescents infected. Chinese pediatricians would like to share their experience on how these patients were managed in China and the key recommendations that had guided them in meeting the evolving challenges. A group of experts were summoned by the Chinese Pediatric Society and Editorial Board of Chinese Journal of Pediatrics to extract informative data from a survey on confirmed COVID-19 pediatric patients in China. Consensus on diagnosis, management, and prevention of pediatric COVID-19 were drawn up based on the analysis of such data plus insights gained from the past SARS and MERS coronavirus outbreaks. Relevant cumulating experiences from physicians managing adult patients, expedited reports on clinical and scientific COVID-19 and SARS-CoV-2 data, and the National Health Committee guidelines on COVID-19 management were integrated into this proposal.

## Introduction

In mid-December 2019, a novel and the seventh human coronavirus strain akin to SARS-CoV was identified (1–3). On January 12, 2019, the World Health Organization (WHO) temporarily named it 2019 novel coronavirus (2019-nCoV). Its high infectivity has contributed to an epidemic spreading to all parts of China and over 100 countries outside China (4). On January 21, 2019, the National Health Commission (NHC) of China defined 2019-nCoV pneumonia as a Class B Notifiable Infectious Diseases to be prevented and controlled by first-level response to major public health emergency. On January 30, 2020, the WHO declared 2019-nCoV epidemic as a Public Health Emergency of International Concern (PHEIC). On February 11, 2020, the International Committee for Taxonomy of Virus (ICTV) officially named the virus as severe acute respiratory syndrome coronavirus 2 (SARS-CoV-2), while WHO named the disease SARS-CoV-2 causes coronavirus disease 2019 (COVID-19). On March 11, 2020, the WHO announced that COVID-19 outbreak has escalated to a pandemic scale.

Riding on the rising peak of COVID-19 pandemic, definitive diagnostic tests were rapidly developed and rendered available to key centers, enabling pediatricians to diagnose and manage the increasing number of COVID-19 cases (5, 6), including severe and neonatal ones (6). Meanwhile, the NHC issued “The Guideline for the Diagnosis and Management Plan for Pneumonia with New Coronavirus Infection (trial 7),” and the WHO released their recommendations via “Global Surveillance for Human Infection With Novel Coronavirus (2019-nCoV),” without much elaborations on the pediatric elements. To address the pediatric specific concerns, the Chinese Pediatric Society (CPS) has initiated a nationwide survey to systematically collect epidemiological and clinical data of pediatric COVID-19. Together with the Editorial Board of the Chinese Journal of Pediatrics, CPS also summoned a multidisciplinary group of experts to put forward recommendations for COVID-19 in pediatrics (7, 8), based on analyzing these data plus experience gained from management of both pediatric and adult cases. The current document does not attempt to assign grading of evidence to the recommendations.

## Clinical Virology of SARS-CoV-2

Four endemic HCoV-229E, -NL63, -OC43, and -HKU1 are prevalent in the general population (9), causing usually mild clinical symptoms, but could be more serious in infants, the elderly, and the immunocompromised (10). In contrast, zoonotic MERS-CoV, SARS-CoV, and the current novel SARS-CoV-2 are opportunistic leading to unexpected outbreaks, with severe pneumonia and even fatality (11, 12). Autopsy and biopsies from adult COVID-19 patients (13) demonstrated SARS-COV-2 RNA and antigen in type II pneumocytes as compatible with *in vitro* findings demonstrating its binding to angiotensin-converting enzyme 2 (ACE2) to enter relevant cell lines. Whether and how opportunistic CoV become endemic is not yet understood, but mutations allowing selection of more adaptive variants capable of surviving better with a human host is one key conceptual mechanism. Ongoing molecular virological studies have already documented SARS-CoV-2 genetic mutants/variants in patients from China (14).

1. Infectious source and prolonged virus shedders:
  Asymptomatic (silent), presymptomatic, and convalescent patients shedding the virus are potential sources of transmission (15–18), especially for adults. Prolonged SARS-CoV-2 positivity was documented in children for at least 4 weeks after onset of symptoms and 2 weeks after being discharged home based on standard guidelines (19). This may carry clinical management implication, as prolonged virus shedding of CoV viruses were found in SARS and MERS convalescent subjects (up to weeks) as well (12), though proof of transmission by convalescent subjects has not been demonstrated.
  With respect to how children participate in the transmission chain of COVID-19, studies from Germany, the United Kingdom, and the United States had provided additional data [on top of those from China (19–23)] defining viral loads across all ages [mainly semiquantitated by the threshold cycles (Ct) data of real-time (RT)-PCR amplification of SARS-CoV-2 genes and less commonly by direct viral cultures] and linking them to rate of transmissibility (17, 24, 25). The suggested correlation of viral semiquantitation (Ct value) to viable virus isolation (26) is particularly important if proven dependable, as it offers non-culture option of determining which SARS-CoV-2-positive individual is a potential spreader of COVID-19 for more evidence-based infection prevention and control (27, 28). Analysis of epidemiological data have all pointed that children are not key primary spreaders of COVID-19 even within family clusters (29–31), which has fueled active debates and also suggestions on how school and daycare centers for children and adolescents should be reopened (32, 33) following the closures during the epidemic.
2. Infectious route
  Direct contact of the mucous membrane of the eyes, nose, and mouth with contaminated hands or infectious droplets (respiratory but also fecal or urinary) is the main mode of transmission. Aerosolization could generate microdroplets to be inhaled (3-μm droplets could remain suspended in air for up to 3 h, while submicron 0.3- to 0.5-μm aerosols could be airborne). Opportunistic transmissions are especially important in enclosed/defined space, known to be enhanced by nebulization therapy or positive pressure mechanical ventilation (5–8, 34). The SARS-CoV-2 virus has been isolated from stool and urine, in addition to respiratory secretions, of COVID-19 patients (35, 36), though fecal–oral transmission has not been definitely proven yet.
3. Epidemiological features
  No specific population is completely spared by COVID-19. As of March 18, 2020, 24:00, there is a cumulative of 81,116 confirmed cases in China, with 70,420 recovered and 3,231 deaths (37). Hubei province, whose capital city Wuhan was the epicenter of this current outbreak, was hard-hit with 67,800 confirmed cases, including 57,678 recovered patients and 3,130 deaths. There were 110,011 confirmed COVID-19 cases reported outside China, involving 161 countries worldwide with 4,576 deaths. Patient age ranged from 36 h to 103 years old, with no gender preference, while those above 60 years or with underlying illnesses exhibited increased disease severity and mortality. Human-to-human transmission was well-illustrated by family and case clusters, together with subsequent exponential growth in cases confirmed highly infectivity of SARS-CoV-2 (5, 6, 37–39). By mid-February mass gathering clusters, including cruise and religious activities, have contributed to outbreaks beyond China.
  The first 10-year-old boy confirmed with COVID-19 was driven from Wuhan to Shenzhen, Guangdong Province, from December 29, 2019 to January 4, 2020, together with five other family members. He was diagnosed and managed there since January 10, 2020 (38). More children were subsequently reported to have COVID-19 (19, 36, 40–44). By March 18, 2020, 720 pediatric confirmed COVID-19 cases have been reported in China, with 27 (all mild) outside of China, by the ongoing CPS survey.
  A total of 714/720 had age data, while 682/720 had gender data. A total of 56.9% (388/682) were boys, and 43.1% were (294/682) girls. Mean age was 7.93 ± 4.26 years with neonates 1.0% (7/714), 29 days−1 year 13.3% (95/714), 1–3 years 14.1% (101/714), 4–6 years 13.2% (94/714), 7–12 years 31.9% (228/714), and 13–17years 26.5% (189/714). They were from 31 provinces, autonomic regions or municipalities in China, the top 6 provinces being Hubei (314 cases), Guangdong (58 cases), Henan (44 cases), Anhui (38 cases), Zhejiang (34 cases), and Shandong (30 cases). For 678 patients with epidemiological exposure information, 23.5% (159/678) had Wuhan-related exposure where SARS-CoV-2 had started spreading, 75.1% (509/678) had definite links to family clusters (38), and 1.5% (10/678) did not have epidemiological exposure history.
  Possible occurrence of vertical maternal–fetal transmission has been supported by two recent reports (45, 46) demonstrating raised anti-SARS-CoV-2 IgM antibodies (in addition to raised IgG antibodies potentially derived from the maternal circulation) in three newborn babies out of seven deliveries from mothers with confirmed COVID-19. These newborns were all asymptomatic. However, reservation about such interpretation of these clinical scenarios has been raised, mainly based on the technical validity of antibodies employed to detect SARS-CoV-2 IgM (47). A retrospective review of nine pregnant women diagnosed in their third trimesters did not have any baby found infected after delivery (48).
  On the other hand, of seven nucleic acid amplification test (NAAT)-confirmed neonatal COVID-19, five were diagnosed during the medical observations postdelivery (with throat/nasopharyngeal swab positive from 36 h to 3 days postdelivery), while the remaining two were diagnosed on postnatal day 17 (PND17) and PND19 after turning symptomatic at home.
  Four early studies had been reported on COVID-19 in children (19, 49–51). One study was conducted in Guangzhou on close contacts of confirmed COVID-19 patients or members from COVID-19 family clusters. It reported a diagnostic rate of 1.3% (10 out of 745) in children based on SARS-CoV-2-specific NAAT (19) using nasopharyngeal swab, with 3.4% (111 of 3,174) in adults. Another study, carried out on all the pediatric admissions in three branches of Tongji Hospital in Wuhan between January 7 and 15, 2020, identified 1.6% (6/366) who suffered from COVID-19 based on nasopharyngeal or throat swab positivity for NAAT (49). The third was on 1,391 hospitalizations in Wuhan Children's Hospital between January 28 and February 26, 2020, which documented 12.3% (*n* = 171) COVID-19 NAAT using throat swabs (50). The fourth analyzed COVID-19 data reported to the Chinese Center for Disease Control between January 16 and February 8, 2020 identified 728 confirmed and 1,407 suspected pediatric COVID-19 cases (51).

## Clinical Features of COVID-19 in Pediatrics

The incubation period for SARS-CoV-2 causing COVID-19 is 3–7 days (medium; range 1–14). Infectious sources are predominantly respiratory secretions (lower > upper in viral load), with saliva, blood, stool, and urine from which SARS-CoV-2 RNA could be isolated. While current clinical guidelines mostly state that COVID-19 patients in clinical remission could be discharged from hospital care when two NAAT performed on relevant specimens at least 24 h apart are negative, this would be revised with more data on virus shedding status coupled with a practical way to determine the viable infectious SARS-CoV-2 biological forms become available.

1. Clinical manifestations
  From the ongoing National Survey of COVID-19 in pediatric population, clinical features were analyzed in 501. Among the 470 patients with body temperature records, 214 cases had detailed data on dynamic changes in temperature, 324 patients of 501 had complete blood count (CBC) tested, while 371 of 501 had chest radiological imaging done.
  Fever (57.9%, 272/470) and cough (39.9%, 200/501) were most frequent, whereas less frequent symptoms included stuffy nose, coryza, sneezing, headache, dizziness, malaise, myalgias, and sore throat. Notably, 53 (10.6%) presented with diarrhea and/or vomiting, a few with abdominal pain, while a 1.5-month-old infant had repeated vomiting only. These clinical symptoms mostly resolved within 1 week. Analysis of 244 consecutive COVID-19 patients admitted into Wuhan Children's Hospital, specifically comparing those with GI symptoms (13.4%) vs. those without (86.6%), showed that the former group was younger (median age 14 vs. 86 months) than the latter (52). See Table 1 for symptom rate.
  Among those with fever, most were short lasting (1–2 days), while 25.2% (54/214) lasted for 3 days or longer, 8.4% (18/214) for 3 days, 7.9% (17/214) for 4 days, 3.5% (9/214) for 5 days, 2.3% (5/214) for 6 days, and 2.3% (5/214) had fever over 7 days with the longest being 14 days in a critically sick child.
  Most CBCs were normal: 5.5% (18/324) children had mild leukopenia (2.96–3.95 × 10^9^/L; one 2.32, another 1.8) and 6.8% (22/324) accompanied by lymphopenia (0.36–1.48 × 10^9^/L; five were <1.0). C-reactive protein (CRP) was normal (<20 mg/L) except transient elevation in 19/142 (9.1%, 20.9–64.6 mg/L) with 47.2 mg/L in one with mycoplasma pneumoniae coinfection and two with pyogenic tonsillitis (51.6 and 64.6 mg/L). A few had mildly elevated liver enzymes and creatine kinase (CK).
  In 371 patients with chest radiological examinations, 235 (63.3%) had ground-glass or interstitial infiltration appearance (including 27 clinically asymptomatic; 4 severe/critical COVID-19, and 3 neonates), 7 (1.9%) only had increased lung markings, while 129 (34.8%) had no radiological abnormality.
  Fourteen patients had evidence of coexposure/coinfection. Eleven were seropositive for mycoplasma pneumoniae IgM among which one had additional Epstein–Barr virus IgM positivity. Three were seropositive for influenza A IgM, while five had respiratory specimens positive for influenza A (2), influenza B (2), and RSV (1) antigens. One of them had prolonged fever (8 days, highest 40.2°C) whose chest CT scan revealed multiple lesions, which resolved completely 4 days later.
  Seven neonates had COVID-19 confirmed. One, diagnosed postnatal day 17 (PND17), presented with sneezing, vomiting, transient fever, and diarrhea; his chest CT revealed bilateral lower lobe reticular changes (44). Another, diagnosed on PND19, was admitted for vomiting and reluctant to feed, then developed fever, cough, and diarrhea with CXR showing bilateral patchy shadows. One only had mild tachypnea and increased lung marking on CXR while another with fever only on PND5. From three reported series of infected pregnant mothers (44, 49), 3 out of the 52 newborn babies prospectively monitored right after deliveries were confirmed to have COVID-19. One was admitted into the NICU immediately (premature 31 weeks 2 days), while the other two were transferred to the NICU on PND1 and 2, respectively (53). The 31 weeker had respiratory distress syndrome, received non-invasive positive pressure ventilation after delivery, and developed disseminated intravascular coagulopathy on PND5, while all three had pneumonia documented by chest CT.
  There were only four pediatric cases requiring mechanical ventilation for respiratory failure. A 13-month-old male who had known bilateral hydronephrosis started suffering from diarrhea, vomiting, and low fever. His fever remitted but resurged by day 7 together with shortness of breath requiring transfer to the ICU. He further deteriorated and evolved to acute respiratory distress syndrome (ARDS), circulatory shock, and acute renal failure (ARF). Chest radiology revealed massive consolidation with ground-glass changes predominately over the right lung. After support by mechanical ventilation and continuous renal replacement therapy (CRRT), he was extubated after 9 days and discharged 17 days after admission (41). The second one was an 8-month-old infant previously operated for congenital heart disease at 1+ months old (ventilated postoperatively for 1.5 months) and suffered from moderate malnutrition. He presented with cough for 6 days then dyspnea for 1 day. He was mechanically ventilated upon admission and successfully extubated 13 days later and eventually discharged 45 days after admission (54). The third case had acute leukemia on maintenance chemotherapy and recovered after supported by mechanical ventilation. The fourth child had background intussusception and developed multiorgan failure, complicating the course, and was the only pediatric fatality associated with COVID-19 (50).
  Based on the clinical classification outlined in the Clinical classification of pediatric infection section below, 230 out of 501 confirmed cases were moderate COVID-19 (45.9%, including 27 subclinical COVID-19 with radiological changes but asymptomatic). Of these confirmed COVID-19, one was severe (0.2%), and four were critical (0.8%). A total of 210 (41.9%) suffered from mild COVID-19, while 56 (11.2%) children were totally asymptomatic with normal radiological findings.
  Out of 219 children with data about their achieving NAAT negativity, 176 cases (80.4%) did so in 1–15 days postdisease onset (DPO1–15), while 29 (13.2%) turned negative by DPO16–20, and 13 (5.9%) did so by DPO21–26. One 3-month-old infant with mild COVID-19 had NAAT negativity by DPO26. The critical COVID-19 8-month-old infant had NAAT negativity of rectal swab on DPO31 and of nasopharyngeal swab on DPO-37, which became positive on DPO38 and eventually negative only by DPO49 (54).
  Compared to adult COVID-19 patients (whose symptoms usually peaked 1–2 weeks after the onset of symptoms, and the duration of virus shedding may persist for 3–4 weeks or longer) (55–57), the pediatric patients had relatively mild symptoms with quick remission and shorter virus shedding duration, except for those with critical COVID-19 and perhaps the young ones. Like in SARS-CoV patients, prognosis is more optimistic in children than adults. Nonetheless, pediatricians should be vigilant and closely monitor each especially those with underlying disorders or above 12 years old, in order to identify whoever is requiring upscaled supportive treatment (51, 58).
2. Clinical classification of pediatric infection
  Based on known clinical features of established pediatric COVID-19 cases, the following classification is proposed (6):
  1. Asymptomatic infection (silent): Patients have NO notable clinical manifestations NOR any radiological abnormality, but SARS-CoV-2 infection is established by positive nuclei acid test or seroconversion.
  2. Mild infection: Patients may present with fever, fatigue, malaise, upper respiratory tract (URT) symptoms like cough, sore throat, rarely running nose and sneezing, or gastrointestinal symptoms like diarrhea, vomiting, and abdominal pain, with physical sign of URTI like pharyngeal hyperemia but not those of LRT.
  3. Moderate infection (regular): Patients have LRT features of viral bronchitis and pneumonia (bronchiolitis is rare) with no tachypnea and hypoxemia, like productive cough, and coarse or moist crepitations evolve. Wheezing is rare. Subclinical variant remains asymptomatic, yet radiologic pneumonic changes are evident (5, 6). There are suggestions that such subclinical variant should not be included (59) in the pediatric COVID-19 classification as radiological imaging (especially CT scan) should not normally be performed on asymptomatic children. Nonetheless, due to the perceived urgency in delineating whether some “asymptomatic” COVID-19 had pathological lung involvements, such investigations had actually been conducted. We opined that this variant should be kept for accurate reflection of real clinical practice, though this should not be undertaken without sound clinical judgment.
  4. Severe infection: These patients usually deteriorate by the second week after onset with respiratory distress, tachypnea, and hypoxemia with SaO_2_ <92% in room air (5, 6, 60, 61); some could have only gastrointestinal symptoms at presentation.
  5. Critical infection: Rarely, a pediatric patient may develop ARDS or respiratory failure. Multiorgan failure, such as circulatory shock, encephalopathy, heart failure, disseminated coagulopathy, and acute renal failure could develop leading to fatality.

**Table 1:** Rate (in percentage) of clinical symptoms in pediatric COVID-19 in China, Italy and USA.

	**Current Chinese Ped Society**	CDC MMWR^*^	Italy CONFIDENCE^**^
Number	501	2,572	100
Fever (>38°C)	57.9	44.0	54
Cough	39.9	54.3	44
Rhinorrhoea/nasal obstruction	9.8	7.2	22
Sore throat	2.6	24.4	4
Shortness of breath	1	13.4	11
Diarrhea ± vomiting	10.5	12.7	9
Headache	2.8	27.8	4
Malaise	4	NA	9
Myalgia	0.8	NA	NA
Chills or rigors	NA	NA	NA

* *CDC COVID-19 response team. Coronavirus disease 2019 in children—United States, February 12–April 2, 2020. MMWR 2020, 69: 422426*.

** *Parri et al. (69)*.

## Laboratory and Radiology Findings

1. Hematological and biochemical test
  1. CBC: Adult patients may have mild leucopenia, lowish absolute lymphocyte counts (with decreased CD4+ and CD8+ subsets), and thrombocytopenia (49, 55, 58). In pediatric patients, total WBC, and lymphocyte and platelet counts were generally normal.
  2. Inflammatory markers: C-reactive protein (CRP) was normal or slightly elevated. Procalcitonin (PCT) was mostly normal. Where available, serum/plasma cytokine and chemokine profiling may help in monitoring COVID-19 disease course and complications (7).
  3. Serum biochemical and coagulation test
    Elevated liver enzyme (ALT and ALT), CK, and myoglobin levels were observed in severe affected children. Some had hypoalbuminemia or coagulopathy reflected by increased D-dimer. Significantly elevated CRP, lactic dehydrogenase (LDH), and serum ferritin could signal or predict deterioration of COVID-19 and related complications (6).
2. Radiology findings
  1. Chest X-ray (CXR): Absence of plain CXR abnormality at surveillance and early symptomatic stage contributed to mild or subclinical COVID-19 patients being missed. In an adult patient, CXR changes are non-specific with bilateral air-space opacities and infiltrates being predominant; effusion or pneumothorax is not common. For non-severe children, CXR changes were compatible with bronchitis, as well as focal patchiness. Diffuse multiple consolidative regions could be observed bilaterally in severe cases. Significant and rapid change in CXR signals potential progression to ARDS.
  2. Chest computed tomography (CT) scan: According to the clinical course of COVID-19, CT changes could be classified into four stages:
    ① Early stage: Subpleural localized consolidation or ground-glass patchiness along the bronchovascular bundles of lung segment or subsegment, with or without concomitant interlobular septal thickening. Unilateral tiny loose opacity in peripheral or subpleural lung field may also be observed.
    ② Evolving stage: Increasing more foci extending to several lobules. Localized consolidation could coexist with ground glass or stripe opacity.
    ③ Severe stage: Bilateral diffuse consolidations, which may progress to classic air bronchogram with “white-out lung.” Pleural effusion or pneumothorax was rare.
    ④ Recovery stage: Resolution of previous lesions (62, 63).
      These radiographic features are not pathognomonic of, nor specific to, CoV infection, but rather reflect the predilection of SARS-CoV-2 (like SARS-CoV and MERS-CoV) to involve LRT. While high-resolution CT scan (HRCT) is more sensitive than plain CXR in delineating pneumonic changes, such modality should be chosen for anticipated semiquantitation and pathogenetic correlation in guiding the choice of non-standard management treatment as most pediatric COVID-19 are mostly mild and self-limiting.
  3. Lung ultrasonography (LUS)
    LUS has steadily gained popularity in pediatric critical care and emergency medicine areas (64). Advantages of LUS include lack of radiation, offering real-time, and point-of-care evaluation either for diagnosing or ongoing monitoring of evolving COVID-19 pulmonary pathologies. Though it has been used in COVID-19 management (65, 66), the urgency and unknown nature of initial COVID-19 outbreak did not allow LUS to be integrated into the Chinese NHC COVID-19 guidelines. Clearly it will eventually be more adopted in managing the ongoing COVID-19 pandemic, as suggested by colleagues in Italy and United Kingdom (67, 68) and had been used in place of CT scan in a series of COVID-19 pediatric patients (69).

## Definite Laboratory Detection of SARS-COV-2 as Pathogen

1. Nucleic acid amplification test (NAAT) with PCR primers specific for SARS-CoV-2 genes is the primary method to detect this pathogen in clinical specimens. Target genes are usually RdRp (RNA-dependent RNA polymerase), E (envelope), and N (nucleocapsid). These tests should be conducted in a P2 health laboratory with operators stringently observing tertiary protection (5–7), but new products potentially suitable for use as points of care tests are emerging.
  1. Sample collection: respiratory specimens is most favorable, including upper respiratory tract (URT) like nasopharyngeal or oropharyngeal swab and low respiratory tract (LRT) like sputum, endotracheal aspirate, or bronchoalveolar lavage. Deep throat saliva is another proven source (20). For strongly suspected cases, a single URT negative test cannot exclude COVID-19 (5, 6, 8). Additional LRT sampling or repeating another URT is recommended. Induction of sputum should be avoided to minimize the risk of airborne transmission (6, 8). Tertiary protection with contact and airborne precautions should be strictly implemented to avoid virus transmitted by droplets from URT or aerosol from LRT (5, 6, 8).
    1.1 For a collection of nasopharyngeal swabs, a synthetic fiber swab with its plastic shaft should be inserted into the nostril parallel to the palate. Pharyngeal swabbing may provoke vomiting, anal swabbing can induce defecation, and both could create hidden environmental contamination and expose healthcare/laboratory workers to profound yet preventable risk. For uncooperative patients, it is prudent to assess whether such sampling is indispensable before committing to doing so (6). It is advisable for workers to conduct these procedures in full PPE. Other specimens such as blood (lower positive rate than respiratory secretions), stool, and urine may be collected, but their diagnostic values should be evaluated prospectively given that relevant information has not been complete (70).
    1.2 For children old enough to clear saliva from deep throat and spit it into the sample collection bottle, detailed instructions on how to do so are available (71).
    1.3 Stool and rectal swab are important samples in working up children especially that there are patients presenting primarily with gastrointestinal symptoms (21).
  2. Laboratory method: Fluorescent real-time quantitative reverse transcription polymerase chain reaction or nucleic acid sequencing to confirm and quantitate presence of SARS-nCoV-2 genome. Full viral genome sequencing is instrumental in identifying variant/mutant SARS-COV-2, which has already been reported (14).
2. SARS-nCoV-2-specific antibody-dependent tests
  1. Serological response could be determined in sera and crucial for confirmation of COVID-19 especially in asymptomatic (silent) patients and those diagnosed by clinical criteria without NAAT positivity. It also enables larger-scale epidemiological studies, screening, and detection of pediatric COVID-19 cases, which are typically mild. More serological tests have been available and used in various reports (72, 73). Similar to the standard diagnostic protocol of SARS-CoV (74), paired serum from acute and recovery phases should be tested for specific antibody titers. Seropositive conversion or greater than or equal to 4-fold elevation in antibody titers can help to retrospectively confirm the diagnosis. Sera-specific antibody-based tests will empower the confirmation of asymptomatic, epidemiological profiling, and detection of pediatric COVID-19 cases (75). As SARS-CoV-2 is a novel human coronavirus, theoretically specific immunity should be absent in unexposed population, and any suspected COVID-19 case found to have a single seropositive sample may be deemed diagnostic. Further investigation on recovery samples to measure the change in titers may strengthen and reaffirm such notion. Of note is that cross-reactivity of less specific antibodies capable of recognizing both SARS-CoV and SARS-COV-2 has actually been a demonstrated issue but could not be totally ignored when working up subjects with unknown infection (76). Given that SARS-CoV infection was not endemic, this should not be a major issue.
  2. Direct viral antigen detection by specific antibodies performed on nasopharyngeal aspirate and other tissues is another standard diagnostic approach. No clinical study has yet been reported for SARS-CoV-2 using such laboratory tests.
3. Virus culture
  The ability to culture SARS-CoV-2 in suitable cell lines (VeroE6/TMPRSS2, Caco-2, Calu-3, HEK293T, Huh-7, and LLC-MK2) (77, 78) offers direct proof whether NAAT-positive clinical samples are indeed infectious. Given the pathogenicity of SARS-CoV-2, stringent guidelines have to be followed by a laboratory doing this and, hence, are not generally carried out. With more comprehensive clinical/laboratory study addressing SARS-CoV-2 nucleic acid test and viral culture positivity, the recovery from the SARS-CoV-2 virus by standard culture has been definitively identified in subsets of symptomatic, presymptomatic, and asymptomatic COVID-19 patients with positive NAAT (17). This corroborates with the clinical observations of asymptomatic COVID-19 patients, who could act as COVID-19 infectors (15–18). Of note is that these studies were conducted principally in adults.

## Diagnosis

Based on the situations of outbreak and the known clinical features of COVID-19 in pediatric patients, we propose to use risk of exposure to assign a patient for medical surveillance, suspected, and confirmed COVID-19.

1. Levels of epidemiology history (EH)
  History of exposure is crucial in early identification and diagnosis of pediatric infection. We recommend defining the history of exposure into three levels (6) within 14 days before the onset of symptoms:
  ① High risk: Those who have close contact with suspected or confirmed COVID-19 subjects, SARS-CoV-2 nucleic acid test-positive subjects, or being a member of a COVID-19 family cluster.
  ② Moderate risk: Those living in an area or community with outbreak of COVID-19 cases.
  ③ Mild risk: Living in an area without confirmed clusters or not highly epidemic.
2. Medical surveillance for COVID-19
  1. All high-risk EH children, regardless of having clinical symptoms or NOT, will be under medical surveillance and have NAAT performed on appropriate specimen(s) at designated center/hospital to avoid missing an asymptomatic patient.
  2. Children with moderate or mild-risk EH should be put under surveillance if one or more of the following are met:
    - Fever
    - Clinical respiratory symptom(s) or others such as fatigue, nausea, vomiting, abdomen discomfort, diarrhea, etc.
      Medical surveillance is traditionally carried out by public health personnel but innovative community networking and tele-platforms have been implemented in Hubei Province, China, to facilitate execution of home-based surveillance to meet unexpected needs (79).
3. Suspected COVID-19
  1. Any neonate born to a mother diagnosed or strongly suspected of COVID-19 (6, 80).
  2. All high-risk EH children OR ANY moderate or mild-risk EH child for whom influenza and other usual pathogens, which could cause his/her clinical symptomatology, are excluded (e.g., clinically non-responsive to oseltamivir treatment for 2 days or by appropriate laboratory tests), who meets two of the following three criteria (6):
    - Clinical—Fever, with obvious respiratory symptom, such as tachypnea or oxygen desaturation or gastrointestinal (GI) presentations such as nausea, vomiting, abdominal discomfort, and diarrhea.
    - Laboratory findings: any decreased WBC count, lymphopenia, and/or elevated CRP.
    - Chest radiological changes compatible with COVID-19.
      All suspected cases need to be quarantined if not hospitalized.
4. Confirmed COVID-19
  Suspected cases will become confirmed once viral nuclei acid, exactly the same or highly homologous to the currently known SARS-CoV-2, is detected in any of the following specimens: nasopharyngeal swab, deep throat saliva, sputum, bronchial lavage, and secretion or clinical specimens from respiratory tract, blood, stool/rectal swab or urine (5–8, 34, 42, 56, 58, 81, 82).
  By mid-February 2020, in view of reported false-negative results (70) and interim limited capacity of NAAT test availability in areas where demands were overwhelming, the NHC included a clinical COVID-19 diagnostic category (ONLY for February 9th to 24th) based on positive epidemiological history plus clinical manifestations plus relevant chest radiological changes with no (or pending) virological confirmation. However, as mild pediatric COVID-19 mainly exhibits minimal, localized, and atypical lung radiological changes (63) not distinguishable from those encountered in simple viral pneumonia especially during the flu season, such clinical category had not been adopted for pediatric COVID-10 care beyond Hubei Province for the specified period.

## Differential Diagnosis

No simple clinical constellations or syndrome is pathognomonic of COVID-19. The common COVID-19 symptoms in children are like those caused by many other viral infections. Suspecting and establishing a rare and opportunistic pathogen like SARS-CoV-2 as being responsible, especially beyond an epidemic outbreak, depend on how known etiological agents could be quickly established or ruled out.

Classic respiratory viruses include, but are not limited to, influenza, parainfluenza, adenovirus, respiratory syncytial virus, rhinovirus, human metapneumovirus, bocavirus, and other endemic human coronaviruses. Other pathogens that could cause pneumonia are mycoplasma pneumoniae, legionella, bacteria, fungus, and tuberculosis. For symptomatic patients requiring hospital admissions, tests for pathogens such as H1N1, H1N2, parainfluenza, RSV, adenovirus, bocavirus, HMPV, rhinovirus, enterovirus (such as EnV-D68), hCoV-229E, hCoV-OC43, hCoV-NL63, hCoV-HKU1, mycoplasma pneumoniae, chlamydia pneumoniae, and legionella could be performed sequentially or even simultaneously according to good basic clinical principles (5, 6, 58, 61, 81, 82). Multiplex rapid PCR-based nucleic acid tests as respiratory or gastrointestinal panels of pathogens are, in fact, regularly used (71). For patients with underlying diseases associated with immune-deficient states, invasive fungal infection should also be considered (1, 4). In case etiological workup for infectious agent is inconclusive, non-infection diseases such as vasculitis, dermatomyositis, idiopathic interstitial pulmonary disease, and cryptogenic organizing pneumonia have to be considered and evaluated (5, 6, 61).

In the context of random emerging serious/new zoonotic viruses, which potentially lead to sinister outcomes or fatalities like H5N1, H7N9, SARS, MERS, and the current SARS-CoV-2, the demand for a wide coverage of known etiological agents is ever-increasing (83). In the community, most viral infections affecting children are self-limiting and hence not traditionally having an exact etiological agent quickly identified and confirmed. When and where resources are available and affordable, speedy point-of-care tests (e.g., rapid antigen test, respiratory pathogen panel by multiplex nucleic acid amplifications) have been increasingly integrated into primary care pediatrician's management of such possible infection(s). Technological advancements rendering these multiplex tests affordable and their clinically relevant turnaround time will help in realizing its contribution to this area (84).

On the other hand, as pediatric COVID-19 is mostly benign, the clinician must avoid being falsely reassured by a diagnosis of COVID-19 as solely or wholly responsible for the clinical symptomatology of an especially sick and toxic child. Good clinical judgment should dictate further exploration for additional cause/coinfection even in the presence of positive laboratory identification of SARS-CoV-2.

## Principle in Clinical Management

1. Basic principle: early detection, early isolation, early diagnosis, and early treatment are the basic principle for management of at-risk children.
  1. Isolate the suspect or confirmed patients as early as possible.
  2. Choose care commensurate with the clinical severity: for asymptomatic or mild cases, especially those residing in an epidemic area where isolation facilities in local hospitals are very limited or not readily available, home isolation and management supported by structured medical surveillance and guidance is a good choice. Nonetheless, hospital care should be more appropriate for those with pneumonia, while more severe and critical cases should be admitted to PICU (6, 42, 58, 82).
2. General clinical management
  1. Isolation
    - Hospitalized patients: Suspected patients should always be cared in a single-patient room until COVID-19 diagnosis is established or refuted. Confirmed cases could be housed together in the same room with appropriate infection control measures observed. Cases with heightened infectivity should be cared in an airborne infection isolation room (AIIR). The health care providers must be equipped according to tertiary protection standard. All biological secretion and excretion from suspected or confirmed COVID-19 patients should be handled according to disinfection guidelines (5, 6, 42, 85).
    - Patients in the community: Condition on available local community health service (*ad hoc* or conventional), the choice of home isolation, and surveillance for a COVID-19 child should be made by the pediatrician in-charge based on the following understandings: his/her parents (or the designated guardians) will accompany the child the whole time; in addition, appropriate community supervising system and tele-medicine guidance pathways are in place and feasible. Specimens for confirmatory diagnostic tests, if deemed appropriate (e.g., deep throat saliva could be self-collected at home), could be sent to, or collected at, designated sites (5, 6, 8, 42, 85).
    - Infants born to a mother with suspected or confirmed COVID-19: Each baby should be immediately separated from his/her mother after delivery and admitted into an isolation room for monitoring. Primary infection prevention should be adopted. Meanwhile, breastfeeding is advised to be temporarily withheld with the mother expressing milk to maintain production for continuing breastfeeding later. Indeed, positive identification of SARS-COV-2 RNA in breast milk had been reported in Italy and Germany recently (86, 87), supporting such notion. It is prudent therefore to check breast milk for NAAT negativity before resuming breastfeeding. Direct breast feeding and nursing by SARS-COV-2-positive mother should be deferred until the mother has viral shedding clearance documented.
      While continual deliberations have been raised as to whether direct contact between nursing mother and her newborn baby should be preferred in the interest of promoting maternal–child developmental health and bonding, the threat of getting moderate/severe COVID-19 is not negligible. The fact that both babies in the Ulm UMC report (87) were infected and became symptomatic (one with hypoxemia) does lend support to the current guideline of separating babies from the mothers.
      The SARS-CoV-2 nuclei acid test for the babies should be performed on respiratory tract and rectal swab samples within 24 h, 5–7 and 14 days after delivery. Any positive result should be reported immediately and further evaluation conducted to inform clinical management, especially to decide whether home isolation and observation is feasible. For home management, all household members must have their COVID-19 status documented before the eligible neonate is discharged. Infection control and prevention measures (with masking, careful disposal of waste, and disinfection) and normal nursing routines of newborn babies will be followed.
      Healthy term infants should stay for at least 7 days; if both 24 h and days 5–7 nuclei acid tests are negative, feeding pattern normal and general conditions good and stable, home isolation and surveillance could be considered. Emergence of any COVID-19 symptom during home surveillance will prompt readmission of the baby for reevaluation. Isolation (at home or in hospital) can only be discontinued at least until postnatal day 14 when NAAT is negative, as decided by the supervising doctor in charge.
      Preterm infants and any term infant with birth asphyxia or other diseases should be isolated in a single room and treated accordingly (6, 80, 88–91).
  2. Clinical supervision: The clinical progress and vital signs will be closely monitored, especially with the use of pulse oximetry to detect hypoxemia promptly. CBC, CRP, PCT, biochemical index (liver enzyme, myocardial kinase, pancreatic enzyme, electrolyte, and renal function), coagulation function, arterial blood gas and urinary test will be performed as deemed appropriate. Chest radiological examination will be determined based on the clinical course (6, 7).
    Clinicians ought to be mindful of possible clinical deteriorations and vigilant over emergence of such clues. Persistent fever, worsening respiratory distress, change of conscious states, cardiovascular compromise, elevated inflammatory factors, and coagulation dysfunction are all important indicators of an emerging critical state, which should be managed without delay (6). Over 50% progression in chest radiological lesions/involvements in any patient should prompt the escalation of his/her care to that of severe and critical cases (see Intravenous immunoglobulin section below).
  3. General and symptomatic treatment should be offered according to clinical needs. Oxygen therapy should be initiated once a patient exhibits dyspnea and/or desaturation (oxygen saturation by pulse oximetry <95%). Oxygen flow rate and mode of ventilation should be carefully chosen and adjusted to achieve sustained optimal oxygenation. Contact precautions should be observed when connecting devices to the oxygen supply (6, 8, 19, 50, 61, 82).
3. Specific therapeutic options
  1. Antiviral treatment: No specific anti-SARS-CoV-2 drug has yet been proven effective. Trials of lopinavir/ritonavir (LPV/RTV), ribavirin, chloroquine, and arbidol treatment have all been proposed for compassionate use according to the Chinese NHC “Diagnosis and treatment plan of pneumonia with new coronavirus infection (trial version 7)” (7). These therapeutic options should be judiciously chosen, generally only for severe or critical cases and preferably only tried/used with informed consent (6). It is prudent for principles outlined in WHO Monitored Emergency Use of Unregistered Interventions Framework (MEURI) (92) to be adopted in choosing a specific agent by the clinical team, with emphasis on structured collection of relevant clinical data for analysis as clinical trials. Along this line, the “Guidance: multisystem inflammatory syndrome temporally associated with COVID-19” issued by the Royal College of Pediatrics and Child Health does illustrate how such proactive approach in dealing with evolving clinical problems is emphasized in the current COVID-19 pandemic (93).
    The second critical COVID-19 child received LPV/RTV for 7 days (started on day 13 and day 6 after onset and admission). His fever subsided after 6 days and was successfully extubated after 7 days; however, his respiratory aspirate remained positive for SARS-nCoV-2 at that time. During therapy, serum triglyceride, total cholesterol, and lactate levels were normal, whereas significantly decreased WBC count and transient thrombocytopenia were observed 3 days after starting LPV/RTV treatment. The leukocytopenia was corrected after a dose of granulocyte-macrophage colony-stimulating factor (GM-CSF) (54).
    On February 17, 2020, Chinese scientists announced that they have found that the antimalarial drug chloroquine has anti-SARS-CoV-2 virus effects (94) and immunomodulatory effects *in vitro*. Through their multicenter clinical studies, chloroquine treatment is demonstrated to be superior than non-chloroquine control by promoting a higher rate of subsidence of fever, alleviating lung pathological changes, decreasing the rate of progression to severe COVID disease, shortening the time for SARS-nCoV-2 nucleic acid positivity converting to negative, and decreasing the overall rate of SARS-nCoV-2 nucleic acid positivity (95). One observational study conducted in New York did not demonstrate either a greatly lower or an increased risk of the defined end point of intubation and death in COVID-19 patients treated with hydroxychloroquine (96). US FDA has issued a review of safety issues with the use of hydroxychloroquine and chloroquine to treat hospitalized patients with COVID-19, which identified reports that included serious heart rhythm problems and other safety issues, including blood and lymph system disorders, kidney injuries, and liver problems and failure. Hence, it has cautioned against the use of hydroxychloroquine and hydroxychloroquine outside the hospital setting or a clinical trial based on the risk of cardia arrhythmia (97).
    Other medications such as arbidol are still under clinical trial (98).
    Usage of antiviral treatment (see Table 2 for dosage recommendations):
    1. LPV/RTV: Extended from the treatment of HIV and dosage in children <40 kg is prescribed according to body weight (99).
    2. Ribavirin: Usage follows those recommended for treating adenovirus pneumonia (100) and better in combination with LPV/RTV.
    3. Chloroquine: According to NHC recommendation (7).
    4. Arbidol is only used in children above 2 years old, based on the Russian National Drug Formulary (98).
      A combination of antiviral drugs is not recommended except for ribavirin. The antiviral agent therapy should be until 24 h following fever subsidence or up to 4 days if deemed ineffective (6). Side effects should be monitored once any antiviral medication is started, and managed or stopped accordingly.
  2. Treatment for severe and critical COVID-19 cases: The guiding principles are an aggressive correction of compromised oxygenation, provision of adequate support to organ functions, and preventing complications.
    1. Respiratory support: For patients with severe hypoxemia and ARDS, respiratory support should be offered by either high-flow nasal cannular or non-invasive mechanical ventilation. If deemed ineffective because of complications like recurrent apnea, ineffective breath, or cardiac arrest requiring resuscitation, invasive mechanical ventilation should be initiated. Sedation, analgesia, and muscle relaxant is required regularly. Tertiary infection control measure should be taken to prevent airborne transmission during the intubating process. Indeed, strategies for promoting health care staff safety during emergency airway management has been outlined (101). Extracorporeal membrane oxygenation (ECMO) is essential if expertise is available. Ideally, the transfer of patients to tertiary centers capable of offering ECMO should best be planned in anticipation (6– 8, 36, 61, 82); hence, background organization in the local health system is often crucial.
    2. Circulation support: Closely monitor vital signs like conscious level, skin color, capillary refilling time, blood pressure, and key parameters including urinary output and lactate level to ensure early identification of septic shock. Septic shock should be treated according to established pediatric guidelines. While fluid bolus therapy is accepted as first-line management, pediatricians should be cautious about potential harm from fluid overload. Preferably accurate evaluation of the patient's hemodynamic status plus careful assessment of its responsiveness following fluid loading should be considered when feasible; however, if that is deemed not feasible, an empiric 20 ml/kg of normal saline could be initiated. Vasopressors should be given early if fluid administration does not restore adequate perfusion. In patients with ARDS, stringent management of fluid intake to achieve a negative balance and treat the syndrome of capillary leakage, while maintaining a normal cardiac and renal function, are crucial. Hemodynamic status should be routinely monitored during the entire course of treatment (6–8, 36, 61, 82).
    3. Multiorgan support: Intensive monitoring of patients for multiorgan function, including neuromuscular, gastrointestinal, urinary, hematological and coagulation systems, and fluid and electrolytes balance. Adopting the Sequential (sepsis-related) Organ Failure Assessment (SOFA) score to semiquantitatively evaluate the function of various systems and determine whether intensive treatment such as CRRT are indicated (36, 61, 82).
    4. Corticosteroid: Corticosteroid has been used in severe COVID-19 patients, like in SARS and MERS, with the understanding that immunopathogenesis contributing to acute lung injuries has been well-documented in macaque SARS-CoV model. New findings have even demonstrated mismatched mounting SARS-CoV spike IgG level and viral loads could be crucial or responsible for such damage (102). It is prudent to believe that rightly timed corticosteroid as an immune-modulator could be beneficial as anecdotally observed in SARS. Both pediatric and adult SARS patients had been studied showing a decrease in raised IL-6, CXCL8, IL-10, and IL-8 by corticosteroid-modulating cytokines with clinical alleviation of lung abnormalities and clinical severity (103–105). Along this line, comprehensive dynamic immune profiles may support selecting the best therapeutic window for halting or preventing life-threatening lung damages.
      In fact, the effectiveness of corticosteroid therapy in reducing mortality in adult critically ill COVID-19 patients have been demonstrated in many studies since the RECOVERY trial first published its positive results in July 2020 (106). The clinical evidence from high-quality RCT studies was evaluated in a meta-analysis led by the WHO “REACT” group (107). Meanwhile, the WHO has actually issued a formal guideline on September 2, 2020, which recommends the use of 7–10 days of systemic corticosteroid therapy in severe and critical COVID-19 patients and conditionally recommends not to do so in the non-severe COVID-19 subjects (108).
      However, as young patients have much milder clinical COVID-19 manifestations, no pediatric-specific corticosteroid therapy trial has been conducted nor reported. We recommend that corticosteroids not be considered in the early phase of disease as it may compromise the virus clearing and inhibit the host's immunity (5, 6, 8), in line with the WHO conditional recommendation. Furthermore, drawing from the experience of adult COVID-19 patients' management (109), corticosteroid may be considered in pediatric patients, based on expert opinions, if three of the following four indications are met: ① fever of more than 38.5°C, lasting for at least 3 days; ② CRP ≥30 mg/L; ③ serum ferritin ≥1,000 μg/L; ④ diffused infiltrative changes documented in both lungs. Presence of these criteria actually indicates that the disease is progressive. The overall treatment period is short, for 3–5 days, which should be discontinued once fever remits.
    5. Intravenous immunoglobulin (IVIG): The efficacy of IVIG therapy in adult patients with COVID-19 is limited. With reporting of multisystemic inflammatory syndrome range of clinical problems in countries outside China, IVIG could well be indicated for confirmed COVID-19 patients with related symptoms, regardless of whether casually triggered by SARS-CoV-2 or not. Therapeutic use of IVIG has been recommended for critical pediatric infection at clinician's discretion (6).
    6. Optimal empiric antimicrobials: Antibiotics should only be used when a coinfection is highly suspected or established by comprehensive microbiological evaluation. Antimicrobial prescription without a conscientious rationale should be avoided. One should ensure its dosing is pediatric appropriate and usage adjusted in accordance to observed clinical response and drug-sensitive test. For patients highly suspected of bacterial coinfection, antibiotics should be initiated immediately while samples be sent for microbiological identification (5, 6, 8, 109).
    7. Traditional Chinese Medicine (TCM): Some recipes have been recommended for adult patients and could be considered by pediatricians who command reasonable knowledge of the respective benefits vs. potential risks or side effects (7). Injectable TCM preparations should be avoided in children (5, 6).
    8. Other treatment (8)
      ① Plasma from COVID-19 convalescent patients with high neutralizing SARS-CoV-2 antibody tire (generally >1:640) had been used as adaptive immunotherapy in severe or critical patients with rapid disease progression (110). Recommended indications are severe or critical patients with rapid disease progression within 3 weeks of clinical illness who have evidence (laboratory or clinical) of SARS-CoV-2 viremia. No pediatric patient has received such therapy in China yet.
      ② Microecological agent: for enhancing balanced microecology of gut microbiomes and prevent emergence of secondary bacterial enteritis.
        See Table 2 for dosage recommendations.

**Table 2:** Dosage of specific therapeutic agents.

	**Route**	**Dosage**	**Frequency**	**Maximal dosage per day**	**Course**
Lopinavir (LPV)	Oral	10 mg/kg	Twice daily	400 mg	1–2 weeks
Ritonavir (RTV)	Oral	2.5 mg/kg	Twice daily	100 mg	1–2 weeks
Kaletra (LPV/RTV)^a^	Oral	Day 14–12 months—LPV/RTV 300 mg/75 mg per m^2^ 16 mg/4 mg per kg	Twice daily		14 days
		>12 months−18 years LPV/RTV 300 mg/75 mg per m^2^	Twice daily	800 mg/200 mg	14 days
		<15 kg−13 mg/3.25 mg per kg			
		>15–45 kg−11 mg/2.75 mg per kg			
Ribavirin	IV	10 mg/kg	8-hourly	500 mg	
Chloroquine	Oral	10 mg/kg	Twice daily	500 mg	
Arbidol	Oral	50 mg (2–6 years old) 100 mg (7–12 years old) 200 mg (>12 years old)	Three time a day		
Methylprednisolone	IV	1–2 mg/kg/day	12-hourly		3–5 days
Convalescent plasma^b^	IV	4 ml per kg	Single dose	200 ml	Single dose but may be repeated if indicated
IVIG	IV	1–2 g/kg	Single dose		May be repeated if indicated
	IV	400 mg/kg/day	Daily		5 days

a *Do not give Kaletra to neonates before 42 weeks postmenstrual age and postnatal age <14 days*.

b *Convalescent plasma should have neutralizing antibody titer >1:640*.

## Case Reporting, Surveillance, Transportation, and Discontinuation of Isolation

1. Detection and reporting
  For patients meeting the criteria to be evaluated for COVID-19, relevant public health ordinance should be followed. These usually translate into activating isolation, prevention, and control measures immediately plus notifying both infection control personnel of respective facility and the local or state health department. If other common respiratory pathogens are undetected, the patient should be sent for SARS-CoV-2 nuclei acid test. COVID-19 may only be excluded by sequential two negative results (with 1 day apart from each other) (5, 6, 8, 42).
2. Medical observation
  A person with close contact or exposure history should be observed and assessed. Centralized or home isolation may be the solution. Duration of the medical observation may last for 14 days from the last contact with the infection source. Temperature and clinical manifestations are the major indicators for upscaling management algorithm (5, 6, 8, 42).
3. Patient transport
  Patients should be transported in a specific ambulance/vehicle with appropriate protection to health care providers. The vehicle should be disinfected after the transportation following the standard practice enlisted in “Transport of Patients with 2019-nCoV Infected Pneumonia (trial version)” (111).
4. Discontinuation of infection control isolation
  Fulfillment of all the following is recommended for discontinuation of isolation: temperature remains normal (not because of ongoing steroid) for more than 3 days, improvement of respiratory symptoms, resolution of lesion in chest radiology, and nucleic acid test for SARS-CoV-2 negative in two successive samples taken more than 1 day apart. The patients may be transferred to a non-isolation ward or discharged home based on clinical judgment (42, 58, 61, 112). Discharged patients should continue to observe home isolation and surveillance for at least another 14 days while wearing a mask, dining separately from other household members, and observe strict hand hygiene. They ought to be promptly re-assessed should any new symptom(s) appear. Follow-up for clearance of SARS-CoV-2 in clinically relevant specimens should be advised by pediatrician in-charge.

## Control of Hospital-Acquired Infection

The regulations implemented by the National Health and Health Commission or other guidelines in patient transport (111–113) and usage of medical protection (42, 85, 114) should be strictly followed. Special attention should be paid to the different risk-level operational regions within a hospital or clinic and the procedures when putting on and taking off protection tools meticulously followed. Infection prevention and control professionals should be clearly designated for health care workers to get clarification of related policies, guidelines, and pragmatic supervision.

## Lessons From Other Countries

With COVID-19 spreading around the globe, more and more pediatric patients had been identified and managed worldwide. This has led to additional key issues being identified, not otherwise covered by the current guideline based mainly on the Chinese experience. An interesting report from Rome, Italy, documented a neonate born to a mother with COVID-19 infection and positive blood SARS-CoV-2 IgG antibody on PND1, but his nasopharyngeal swab NAAT only turned positive by PND14 (negative on both PND1 and 3) (86). He remained asymptomatic. Whether the presence of maternal SARS-CoV-2 had protected him from turning symptomatic remained to be speculative. Interestingly, SARS-CoV-2 had been identified in the breast milk (actually the first report in medical literature) in the early postnatal days. In this report, abstinence of breastfeeding until the maternal COVID-19 infectious status cleared was practiced, in agreement with our recommendation. In order not to deprive these newborn babies of the crucial benefits of breastmilk feeding, donor breast milk, or those from the milk bank could be considered (115). Furthermore, elaborate long-term follow-up protocol up to 1 year of age has been proposed for those newborn babies with established COVID-19, which is worth following in order to fully delineate the clinical impact of COVID-19. Meanwhile, how best should newborn babies of confirmed pregnant mothers be managed will continue to be refined with more experiences cumulated worldwide.

On the other hand, strikingly different clinical syndromes associated with COVID-19 had been reported in pediatric patients outside Asia, namely, hyperinflammatory shock syndrome, multisystem inflammatory syndrome, Kawasaki-like syndrome, atypical Kawasaki syndrome, Kawasaki disease shock syndrome, and toxic shock syndrome (116–118). This unexpected range of clinical manifestations has not been encountered/reported in China (including Hong Kong Special Administrative Region and Taiwan) even after a retrospective review of known COVID-19 patients for related features. No uniform explanations have been reached yet.

With the looming threat of missing pediatric COVID-19 patients taking acute downturn during the course of illness, Chinese pediatricians are more inclined to use CT scan to definitively delineate their possible COVID-19 pulmonary involvements speedily. Now that pediatric COVID-19 was found to be mostly taking self-limiting courses, protecting asymptomatic (mainly close contacts of confirmed cases) and mild symptomatic subjects against avoidable exposure to high radiation doses of CT scans by adopting an alternate algorithm involving expanded use of LUS, becomes very appropriate (119).

With the health care service models being widely diversified across the world, variations seen in guidelines of de-isolations were primarily linked to the respective national and state epidemic status and resources. The US CDC has reviewed the details pertaining to the evolution of the situation plus the cumulated evidence and shared on its website, e.g., the Discontinuation of Transmission-Based Precautions and Disposition of Patients with COVID-19 (120). Likewise, the European CDC has issued comprehensive documents for pragmatic guidance, e.g., discharge criteria for confirmed COVID-19 cases (121).

## Concluding Remarks

Following the emergence of the third zoonotic coronavirus SARS-CoV-2 infecting humans with a clinical syndrome marked by serious pneumonia and potential fatality like SARS and MERS, over 100,000 patients have COVID-19 confirmed with over 3,600 deaths in a short 2.5 months. Despite there are yet no definitive treatment(s) proven by clinical trials to cure these coronaviral diseases, anecdotal experiences and basic scientific studies from SARS and MERS outbreaks have lined up leading agents and thoughts adopted by critical care clinicians. A few tangible antiviral and anti-inflammatory treatments were being used in severe or critically ill COVID-19 patients, with mixed claims of effectiveness. These were constantly reflected in the various updated versions of the National Health Committee guidelines for COVID-19 management.

With endemic coronaviral infections all relatively benign, the milder clinical course observed in the current pediatric COVID-19 will again not be conducive to much planned pediatric-specific studies for understanding crucial elements rendering childhood coronaviral infections unique. Arguably, the choices of management and treatment for pediatric subjects could well be distinct from adults, particularly in the rare occasions of more severe complications. For example, serious side effects previously reported in adult SARS patients from high-dose steroid usage were not experienced by the pediatric counterparts, while beneficial effects were apparent (122). Getting to better delineate what pediatric COVID-19 is like will set the stage to establishing informed management approach, allowing stakeholders to identify targeted agenda to explore for plausible differential treatments fit for our developing kids.

## Author Contributions

All authors listed have made a substantial, direct and intellectual contribution to the work, and approved it for publication.

## Conflict of Interest

The authors declare that the research was conducted in the absence of any commercial or financial relationships that could be construed as a potential conflict of interest.
